# Kinematic “4 Dimensional” CT Imaging in the Assessment of Wrist Biomechanics Before and After Surgical Repair

**Published:** 2013-02-23

**Authors:** Jaimie T. Shores, Shadpour Demehri, Avneesh Chhabra

**Affiliations:** ^a^Department of Plastic and Reconstructive Surgery; ^b^Russell H. Morgan Department of Radiology and Radiological Science, The Johns Hopkins University School of Medicine, Baltimore, Md

## Abstract

**Aim:** Describe the use of 320 row detector CT scanner for 4 Dimensional CT acquisition on a specialized platform designed for wrist kinematic evaluation and to demonstrate the utility of 4 Dimensional CT in the assessment of wrist biomechanics before and after surgical repair. **Materials and Methods:** Six wrists (1 volunteer and 4 patients) were uniformly imaged with conventional X-rays and 4 Dimensional CT on a 320 row detector CT scanner (Aquilion one, Toshiba, Tokyo, Japan). A dedicated custom designed wrist platform was used for kinematic imaging. Three subjects (3 wrists) had prior fixation of the complex wrist injury. Clinical correlations were obtained. **Results:** All subjects were successfully scanned in various wrist motions. 4 Dimensional CT image quality was adequate and carpal kinematic behavior was easily assessed in various wrist motions both before and after surgical repair. The normal and altered carpal kinematic behaviors correlated well with the clinical findings. In the operated wrists, while X-rays demonstrated slight gapping after scapholunate ligament repair, kinematic imaging demonstrated no abnormal widening on dynamic motion and showed normal dorsally hinged “scissoring” type scapholunate motion with active wrist motion. However, normal mid-carpal initiated wrist motion on flexion/extension was replaced by radiocarpal initiated motion, likely because of midcarpal stiffness/scarring. **Conclusion:** 4 Dimensional CT provides adequate and novel assessment of wrist biomechanics both before and after surgical repair.

The human wrist, which is central to the majority of activities of daily living, is a complex arrangement of multiple, small bones, which allows for a significant degree of physiologic motion. Twenty-eight percent of all injuries to the musculoskeletal are hand/wrist injuries^1^ and the prevalence of wrist pain in all athletes is approximately 8%.^2^ The inherently unstable carpal structure is balanced by a hierarchy of primary and secondary ligaments.^3^^-^9 In the healthy state, there are no substantial differences in the dynamic motion and stepwise static alignment of the carpal bones.^10^ However, pathology and injury (eg, acute/chronic trauma, arthritis, synovitis, and depositional diseases) can imbalance this complex ligament structure, enabling subtle alterations in the direction and degree of the hand position that ultimately changes the kinematic behavior of the individual carpal bones.^11^^-^14 Often this balance disruption is irreversible, causing distortions in force transmission and setting the stage for progression to permanent wrist pathomechanics, chondral loss, and bony productive changes.^15^ Preventing this progression by reestablishing the healthy ligament balance has been hindered by inadequate clinical diagnostic tools and a lack of knowledge with regard to the exact epidemiology of this spectrum of instabilities. Even after surgical repair or reconstruction, wrist injuries ranging from isolated scapholunate (SL) ligament tears to Mayfield Grade IV lunate dislocations may progress on to symptomatic or asymptomatic instability and arthritis. Conventional X-rays, fluoroscopy, or static CT are usually performed to guide postoperative rehabilitation and function. However, dynamic imaging has not been described yet as a tool for assessing adequacy of ligamentous stability or normalcy of intercarpal motion due to technical demands and expertise needed in its interpretation.

## MATERIALS AND METHODS

**Figure 1a, b:**
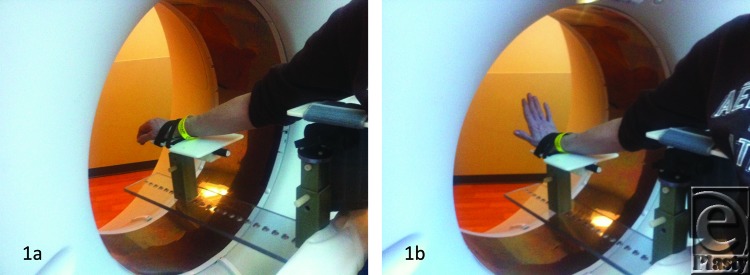
Dedicated “in CT” wrist platform. Pictures taken during unconstrained flexion and extension motions.

“4 Dimensional” CT kinematics were obtained on 6 wrists, 1 volunteer and 4 patients (both wrists in 1 patient) (Table 1). All subjects were uniformly imaged with conventional radiographs (frontal and lateral views) and 4D CT on a 320 row detector CT scanner (Aquilion one, Toshiba, Tokyo, Japan). A dedicated custom-designed wrist platform (Fig 1) was used for kinematic imaging that allowed in-scanner unconstrained wrist motions in different directions during gantry rotation and scanning. All subjects were pretrained for various movements by a research fellow, and each motion was completed in approximately 5 seconds. Following a successful scan on a volunteer, another 4 patients were imaged. Three subjects (3 wrists) had prior fixation of the complex wrist injury. Open reduction and internal fixation of the distal radius fracture was performed along with bone anchor repair of scapholunate interosseus ligament injury and a dorsal wrist capsulodesis utilizing a 4-mm slip of dorsal intercarpal ligament as described by Szabo. Percutaneous 0.045 k-wires were used to fixate the scapholunate and scaphocapitate joints for isolated scapholunate interosseus ligament (SLIL) repairs and additionally lunotriquetral and triquetrohamatocapitate pins were placed for Mayfield Grade III and IV injuries. K-wires were cut short and buried beneath the skin and kept in place for as long as tolerated by patients with a goal of 12 weeks. Complete wrist immobilization within a cast or locking brace was maintained until the removal of k-wire. Once removed, motion was instituted 2 weeks later in supervised hand therapy. Dynamic CT imaging was then performed after initiation of wrist motion. A combination of various motions were performed in the subjects namely, radioulnar deviation, flexion-extension, supination-pronation, clenched fist maneuver, and dart-throwing motion leading to 15 to 20 seconds scanning time for each wrist as described in Table 1. 4D CT reconstructions were uniformly obtained by the research fellow using in scanner software and were loaded onto the conventional PACS (picture archiving and storage system, UV Emageon, Alabama). Both X-rays and 4D CT imaging findings were reported by a musculoskeletal radiologist (A.C.—15 years of radiology experience), including the image quality and normal as well as altered wrist motions. The imaging findings were correlated with the clinical findings.

## RESULTS

**Figure 2a, b:**
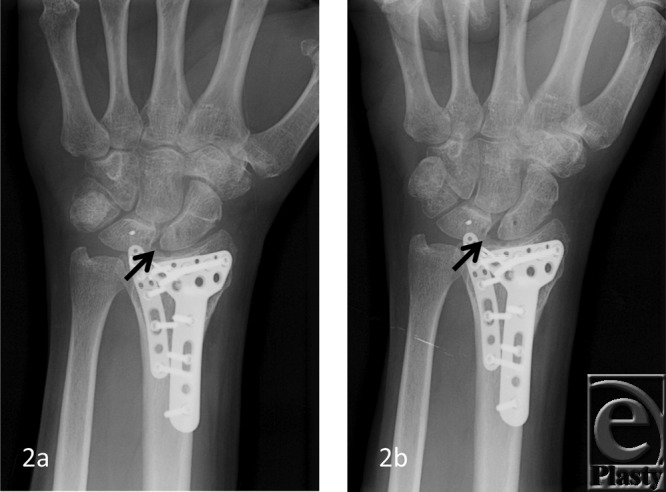
A 62-year-old man with prior ORIF and SLIL ligament repair. Normal SL space (*arrow*) on posteroanterior X-ray view (*a*). Notice mild gaping of S-L interval (*arrow*) on clenched fist posteroanterior X-ray view (*b*). ORIF indicates open reduction internal fixation; SLIL, scapholunate interosseus ligament.

**Figure 3a, b.**
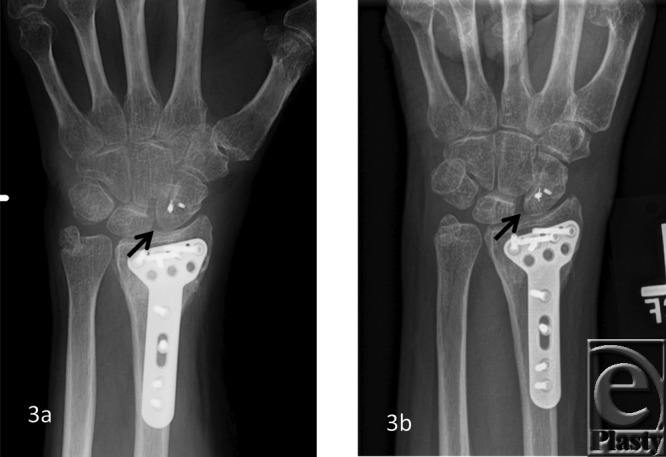
A 50-year-old woman with prior ORIF and SLIL ligament repair. Normal SL space (*arrow*) on posteroanterior X-ray view (*a*). Notice mild gaping of S-L interval (*arrow*) on clenched fist posteroanterior X-ray view (*b*). ORIF indicates open reduction internal fixation; SLIL, scapholunate interosseus ligament.

**Figure 4:**
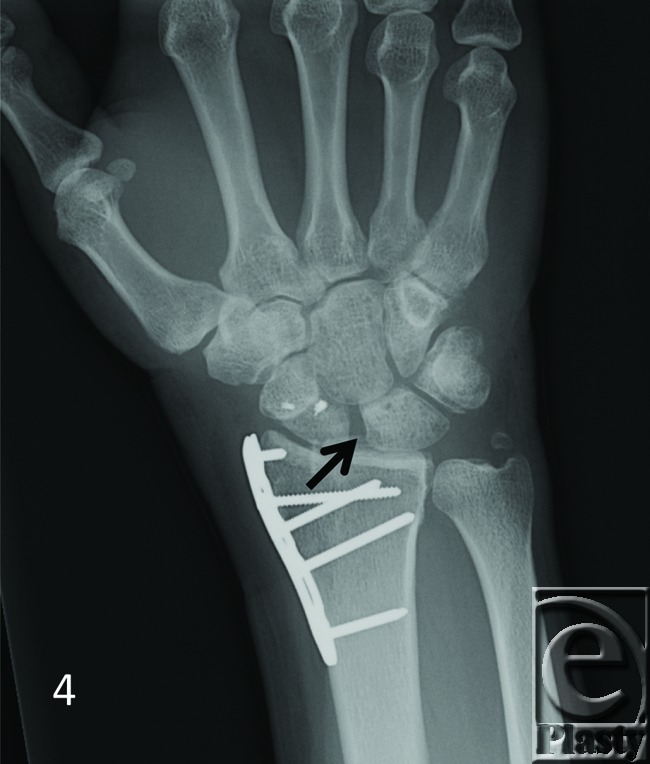
A 30-year-old man with prior ORIF and SLIL ligament repair. Notice mild gaping of S-L interval (*arrow*) on conventional posteroanterior X-ray view. ORIF indicates open reduction internal fixation; SLIL, scapholunate interosseus ligament.

**Figure 5:**
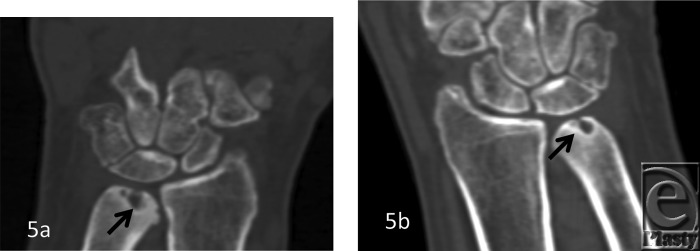
A 45-year-old man with diffuse wrist pain. Outside plain films not available. 2D reconstructions from the kinematic 4D CT volume scan show ulnar sided carpal translocation bilaterally. Also note subchondral cystic changes of ulna bilaterally (*arrows*) suggesting dynamic ulnocarpal abutment.

The patient demographics, clinical findings, X-ray, and 4D CT imaging findings as well as final patient disposition are depicted in Table 1. All subjects were successfully scanned in various wrist motions (Patient 1-4 videos). 4D CT image quality was adequate in all subjects, and carpal kinematic behavior was easily assessed in various wrist motions both before and after surgical repair. The normal postoperative (Patient 1-3 videos) and altered carpal kinematic behaviors (Patient 4 videos) correlated well with the clinical findings. In the operated wrists (Figs 2-4), while X-rays demonstrated slight gaping after scapholunate ligament repair, kinematic imaging demonstrated no abnormal widening with dynamic motion and showed normal dorsally hinged “scissoring” type scapholunate motion with motion. However, normal mid-carpal initiated wrist motion on flexion/extension was replaced by radiocarpal initiated motion, likely because of midcarpal stiffness/scarring. Clinical suspicion of ulnocarpal abutment was also confirmed in both wrists of Patient 4 (Fig 5).

## DISCUSSION

The human carpus is a complex system of 7 load sharing bones plus a sesamoid (pisiform) linked between the distal radius and ulna and the metacarpal bases with various static (ligament) and dynamic (musculotendinous units) stabilizers. Conventional static X-rays are used to follow patients longitudinally after injury, along with symptom development and physical examination, to determine whether or not a patient may have a ligamentous injury requiring intervention. The early insensitivity of static radiography for scapholunate ligament instability is well known.^16^ Standard findings of a “DISI” deformity—obtuse scapholunate ligament angle, loss of collinear lunocapitate angle, scapholunate diastasis (“Terry Thomas” sign), or a flexed scaphoid “ring sign”—may not develop for weeks or months after a complete injury to the dorsal limb of the SLIL and has also been associated with the need for not only an injury to the SLIL but also the dorsal intercarpal ligament.

Patients with wrist instability variably present with symptoms of pain, wrist clunk, and decreased grip strength similar to patient 4. Many a times, SLIL or LTIL (lunotriquetral interosseus ligament) injuries may not be fully appreciated until static signs of injury occur weeks to months or longer after an injury. This may diminish the possibility of standard repair of the injury and instead result in the need for more complex and invasive reconstructions or the development of irreversible arthrosis. In the absence of clear radiographic and/or physical examination indicators, magnetic resonance imaging with or without arthrogram, or wrist fluoroscopic evaluation with arthrogram, may be obtained to aid in diagnosis. However, even a partial injury may show positive findings with arthrogram, and the sensitivity of magnetic resonance imaging may vary. In addition, asymptomatic ligament tears are commonly seen on MR imaging or MR arthrograms.^17^ Although planar stress views and videofluoroscopy has been used to evaluate carpal kinematics,^18^^-^20 the measurement of intercarpal angles is difficult and subject to a great degree of variability between examiners.^3^ The ligament sectioning using 3D animated modeling has been shown to improve the accuracy of detection of intercarpal widening and angular deformations by avoiding overlap among the bony contours.^21^^-^27 Yet, these studies have been limited to in vitro models and are not directly comparable to in vivo kinematics because of complex motions which occur during in vivo tendon excursions.^19^^,^^20^^,^^28^

Our understanding of the biomechanics involved in allowing stable motion with and without loading continues to develop. Its evolution has progressed substantially from a tightly linked ball-and-socket type joint to the central column theory described by Navarro^29^ and later Taleisnik,^30^ to the oval-ring concept proposed by Lichtman,^31^ which is most currently favored. While our understanding of the bony and ligamentous anatomy seems satisfactory at this point, we continue to learn more about extrinsic stabilizers and how afferent innervation of the known wrist ligaments works with effector muscles, such as, the extrinsic forearm flexors and extensor to stabilize a wrist in motion and under load. However, most biomechanical studies detailing radiocarpal and midcarpal motion are performed on cadaveric specimens devoid of this normal stabilization system of extrinsic muscles, which are just now being appreciated. Motion is studied in this manner without regard to normal protective mechanisms that may limit motion to diminish strain in certain innervated ligaments and thus joints. Whether or not this results in motion with active use that is different than that studied in a cadaveric biomechanical laboratory is unknown. Thus, even using a combination of clinical tests, false-negative results remain common, which result in, continued patient suffering, worsening disability, and mounting treatment costs. Therefore, there is clearly a need for more accurate and precise, in vivo evaluation of carpal bone kinematics in patients presenting with symptoms of instability.

Spiral and multislice imaging built great milestones in the history of CT. 4D kinematic CT offers the next leap forward in CT technology that allows acquisition of isotropic volumes of an entire wrist with a single rotation of the gantry. The ability to acquire the entire wrist from distal forearm to metacarpal heads with one volume scan opens the door to new diagnostic possibilities and can revolutionize patient care. It allows real time visualization of the wrist dynamics during various unconstrained motions, such as supination-pronation, flexion-extension, fist clenching, and radialulnar deviation/dart throwing motions.^32^^,^^33^ A rapid 15- to –20-second study on a 320 slice scanner provides excellent depiction of the wrist motion relative to the forearm and concomitant visualization of the intercarpal translational as well as angular relationships. It was possible in all of our cases with adequate diagnostic image quality. Analyzing in vivo joint motion in such a manner avoids the need for invasive marker techniques.^18^^,^^19^

Patients with obvious findings necessitating surgical intervention such as the radiographic findings already mentioned, or more complex injuries such as a perilunate or lunate dislocation, may have questionable radiographic findings even after surgical repair/reconstruction. A persistent 3- to 4-mm scapholunate gap suggesting persistent ligamentous instability despite repair on multiple unchanging postoperative X-rays following fixation removal can be common finding that delays initiation of wrist motion rehabilitation and return to normal function, as it was observed in patients 1 to 3. For these reasons, we have begun incorporating Dynamic “4D” CT imaging into our postoperative imaging following commencement of wrist motion and rehabilitation for patients with interosseus ligament injuries of the wrist. We could obtain good diagnostic quality imaging and 4D reconstructions in all subjects without significant beam hardening artifacts similar to earlier results on 256-row detector study.^34^ For this modality to have its highest diagnostic sensitivity and specificity, it requires a thorough knowledge of wrist kinetics to appropriately appreciate what motion is normal and what motion is abnormal. This requires collaboration of hand surgeons and radiologists with study of “normal” dynamic studies and comparison with “abnormal” studies. These cases represent our early experience with this modality and how it has assisted in appreciating the postinjury wrist motion as well as helped guide activity and treatment postoperatively.

The ability to rapidly deliver high-resolution images has made CT a core imaging modality across healthcare today. However, there have been increasing concerns over patient safety due to radiation exposure and ALARA (As Low As Reasonably Achievable) principle should be followed. The extended coverage provided by the 16-cm wide area detector enables high-quality scanning of the entire wrist within one rotation, eliminating the need for helical scanning, which in turns lowers the dose dramatically. Although the extremities are more exposure resistant, there are a number of approaches used in these scans to reduce exposure dose to the patient. These include active collimator, AIDR 3D (Adaptive Iterative Dose Reduction 3D), SUREExposure 3D (automated exposure control) and quantum denoising software. The Active Collimator prevents extra radiation dose by eliminating rays that are not used for image reconstruction. It operates automatically at the start and end of scan range limiting the extent of the X-Ray beam. AIRD 3D is an advanced iterative reconstruction algorithm used in our scanner that reduces noise in both the 3D reconstruction data and raw data domains. SUREExposure 3D is another function that continuously modulates the exposure dose (by altering mA) in all 3 directions based on the patient's wrist shape. With the integration of AIDR 3D into SUREExposure controls, radiation exposure is automatically reduced before the scan, ensuring that the lowest possible dose is employed for the specific diagnostic objective irrespective of the size or shape of the patient. For routine clinical use, with the use of these functions, one is able to remove up to 50% of image noise that corresponds to a dose reduction of up to 75%, ultimately leading to improved spatial resolution and excellent artifact (streak/beam hardening) reduction that are otherwise routinely present at low dose acquisition. There is also minimal penalty in reconstruction times. Quantum Denoising Software is a powerful tool to remove noise in reconstructed images. It works in all dimensions and selectively smoothens out areas of uniform density while preserving the edge information within the image. It also reduces noise and increases the signal-to-noise ratio, while preserving contrast resolution. Finally, Boost3D is a 3D technology that automatically detects streak artifacts, and it eliminates them by reducing the photon starvation effects in the raw data domain. As a result, the image quality is improved in all viewing planes. A technologist training program ensures that operators become proficient in taking advantage of all the dose-reduction technologies available for every scan, providing clinicians with maximum image quality at the lowest possible dose to the patient. We achieved a dose of CT dose index of 10.5mGy and a dose length product of 84.3mGy-cm, close to 2× to 3× that of a static high-resolution scan. The effective dose was only 0.07mSv per scan maneuver (Table 2). With use of low-dose imaging, this novel modality provides an ability to evaluate the effect of muscle loading as well as long-term effects of treatment.^28^^,^^35^ Dynamic evaluation of the carpus promises to be a useful tool when trying to assess for adequacy of repair or reconstruction of complex wrist injuries. Using ALARA principle and the dose-reducing methods described earlier, 4D CT can be used for longitudinal studies without worrying about excessive radiation risks.

This study had certain limitations. In this pilot study, we did not do reader reliability evaluation or direct comparison of scan quality between 2D, 3D, and 4D reconstruction images, with static high-resolution scan. We did not perform any computational analysis for detection of subtle abnormalities and we only relied on gross visual evaluation of abnormality by the experienced reader. Further longitudinal study is warranted with this technique and should include preoperative and postoperative evaluation of ligamentous injuries in the same subjects for the evaluation of incremental value of this technique over conventional CT examination.

To conclude, kinematic 4 dimensional computed tomography (4DCT) provides adequate and novel assessment of wrist biomechanics both before and after surgical repair.

## Figures and Tables

**Table 1:** Patient characteristics

Subject	Age, y	Sex (M/F)	Prior Surgery	Symptoms at Time of 4DCT	X-ray Findings	Clinical Question	4DCT Maneuvers	Relevant Findings	Final Disposition
Volunteer	18	M	None	None	None	None	F-X S-P R-U	Normal	Normal
Patient 1	62	M	ORIF of distal radius fracture and SL ligament repair	Normal postoperative stiffness, no pain	Mild SL diastasis on supinated clenched fist view	? SL instability	F-X S-P Dart	No kinematic instability	1 year follow-up from surgery-well healed with no instability
Patient 2	50	F	ORIF distal radius fracture and SL ligament repair	Normal postoperative stiffness, no pain	Mild SL diastasis on supinated clenched fist view	? SL instability	F-X S-P Dart	No kinematic instability	4 months follow-up from surgery-well healed with no instability
Patient 3	30	M	ORIF distal radius fracture and SL ligament repair	Normal postoperative stiffness, no pain	Mild SL diastasis on supinated clenched fist view	? SL instability	F-X S-P Dart Clenched fist	No kinematic instability	6 months follow-up from surgery-well healed with no instability
Patient 4 Right Wrist	45	M	None	Diffuse wrist pain, worse with motion	Ulnar subluxation of carpus on outside X-rays	? Ulnocarpal impaction	F-X R-U Clenched fist	Dynamic ulnocarpal abutment on radial deviation and relaxed phase of clinched fist motion	Surgical fixation planned
Patient 4 Left Wrist	45	M	None	Diffuse wrist pain, worse with motion	Ulnar subluxation of carpus on outside X-rays	? Ulnocarpal impaction	F-X R-U Clenched fist	Dynamic ulnocarpal abutment on radial deviation and relaxed phase of clinched fist motion	Surgical fixation planned

Dart indicates darth throwing motion; F-X, flexion-extension; ORIF, open reduction internal fixation; R-U, radial-ulnar deviation; S-P, supination-pronation; SL, scapholunate; ?, questionable.

**Table 2:** 4D kinematic CT wrist scanning parameters compared to static high resolution 3D scan

Scan Mode	Slice thickness	Range	kV	mA	Rotation Time	Acquisition Time	Acquisition Interval	CTDIvol (mGy)	DLP (mGy-cm)	Effective Dose
Static high-resolution 3D scan										
Volume	0.5 mm × 160	80 mm	120	100	0.5 s	NA	NA	4.1	32.4	0.67
4D Kinematic Dynamic Volume (Large patient or with metal in situ)										
Dy-Volume	0.5 mm × 160	80 mm	100	100	0.5 s	5.5 s	Continuous	26.0	207.7	0.17
4D Kinematic Dynamic Volume (Average patient)										
Dy-Volume	0.5 mm × 160	80 mm	80	80	0.5 s	5.5 s	Continuous	10.5	84.3	0.07

NA indicates not applicable; CTDIvol, CT dose index volume.

**Figure:** **Patient 1, Videos 1-3** Video 1. Flexion-extension maneuver: Notice no dorsal intercalated segmental instability (DISI), volar intercalated segmental instability (VISI), or midcarpal instability. Hardware colored in green. **Video 2.** Dart throwing maneuver in semitransparent see-through view: Normal carpal gliding motion with no disruption in carpal arcs or abnormal S-L space widening. **Video 3.** Dart throwing maneuver in surface display: Normal carpal gliding motion with no disruption in carpal arcs or abnormal S-L space widening.

**Figure:** **Patient 2, Videos 1-3.** • Video 1. Dart throwing maneuver: Normal carpal gliding motion with no disruption in carpal arcs or abnormal S-L space widening. **• Video 2.** Flexion-extension maneuver: Notice no DISI, VISI, or midcarpal instability. **• Video 3.** Supination-pronation maneuver: Normal congruent distal radioulnar joint motion.

**Figure:** **Patient 3, Videos 1-3.** • Video 1. Dart throwing maneuver: Normal carpal gliding motion with no disruption in carpal arcs or abnormal S-L space widening. **• Video 2.** Flexion-extension maneuver: Notice no DISI, VISI, or midcarpal instability. **• Video 3.** Clenched fist maneuver: No abnormal scapholunate widening.

**Figure:** **Patient 4, Videos 1-3: Right wrist.** • Video 1. Radioulnar maneuver: Ulnocarpal abutment evident on radial deviation position. **• Video 2.** Flexion-extension maneuver: Notice no DISI, VISI, or midcarpal instability. **• Video 3.** Clenched fist maneuver: No abnormal scapholunate widening. Ulnocarpal abutment seen on relaxed phase disappears on clenched fist.

**Figure:** **Patient 4, Videos 4-6: Left wrist.** • Video 1. Radioulnar maneuver: Ulnocarpal abutment evident on radial deviation position. **• Video 2.** Flexion-extension maneuver: Notice no DISI, VISI, or midcarpal instability. **• Video 3.** Clenched fist maneuver: No abnormal scapholunate widening. Ulnocarpal abutment seen on relaxed phase disappears on clenched fist.
